# Synthesis and Characterization of High Viscosity Cationic Poly(Proline-Epichlorohydrin) Composite Polymer with Antibacterial Functionalities

**DOI:** 10.3390/polym14142797

**Published:** 2022-07-08

**Authors:** Mithil Kumar Nayunigari, Rominder Suri, Gangadhar Andaluri

**Affiliations:** Department of Civil and Environmental Engineering, Temple University, 1947 North 12th St, Philadelphia, PA 19122, USA; tuj48880@temple.edu (M.K.N.); rsuri@temple.edu (R.S.)

**Keywords:** cationic polymer, electrostatic interaction, Gram-negative microorganism, adsorption, congo red, microbial inactivation

## Abstract

We report microbial resistance and catalytic activity of high viscosity cationic poly(proline-epichlorohydrin) composite (PRO-EPI) in the aqueous system. The PRO-EPI was prepared by a simple polycondensation, followed by FTIR, ^1^H NMR, SEM, DLS, viscosity, and DSC/TGA characterization. Several concentrations of the PRO-EPI were tested against Gram-negative (*E. coli* and *Pseudomonas aeruginosa*) and Gram-positive (*Staphylococcus aureus*) microorganisms. The antimicrobial screening revealed that PRO-EPI was a potent antimicrobial agent with the least inhibitory concentrations (MICs) of 128 µg/mL against Gram-negative microorganisms. The PRO-EPI indicated no inhibitory effect against Gram-positive microorganisms. It was determined that PRO-EPI contains polymeric-quaternary ammonium compounds that inactivate the Gram-negative microorganisms by a dual mode of action and carries domains for electrostatic interaction with the microbial membrane and an intracellular target. To study the removal of toxic industrial wastewater, congo red (CR) was tested using sodium borohydride as a reducing agent. Adsorption was achieved within 20 min at a rate constant of 0.92 ks^−1^. UV–vis spectra showed that the removal of CR in the reaction solution was due to the breakup of the azo (–N=N–) bonds and adsorption of aromatic fragments. PRO is biodegradable and non-toxic, and PRO-EPI was found to be both antimicrobial and also acts as a catalyst for the removal of congo red dye.

## 1. Introduction

Several chemicals such as iodophors, inorganic salts, organo-metallics, phenols, thiophenols, heterocyclics, urea, nitro compounds, and formaldehyde by-products are known for their antimicrobial activity. However, most of them are toxic and not readily biodegradable in the environment [1]. Various types of antimicrobial agents, disinfectants, antibiotics, and antiseptics are traditionally used to efficiently combat microbial pathogens [2]. However, drug resistance to commonly used antibiotics has become widespread, and the slow development of new antibiotics has increased the threat to public health [3,4,5,6,7]. Conventional antimicrobials were proposed based on their low molecular weight; unfortunately, these agents are easily prone to resistance and result in contamination and probably impart toxicity to humans due to their biocidal dispersion [8,9]. Therefore, the synthesis of compounds that could be used for the efficient treatment of communicable diseases without any side effects is a crucial biomedical requirement [10]. Several compounds have shown antimicrobial potential, including silver nano materials, polymers that contain nuclei that promote antimicrobial activity as well as reduce residual toxicity [11,12,13,14,15,16,17]. Likewise, antimicrobial polymers retain chemical stability, demonstrating long-term activity [18]. Realizing the medicinal importance of polymers, it was considered worthwhile to incorporate biologically active molecules into synthetic polymer moieties [19,20]. Consequently, in this work, we synthesized polymers with the goal of effectively enhancing antimicrobial activity.

Positive charges of cationic polymers attached to surfaces of microbes were used as antimicrobial agents in sophisticated formulations or directly by themselves [21]. Among these, the polymeric materials containing quaternary ammonium compounds (QACs) were widely used [2] and were developed as capable materials for further development as antimicrobial agents [21,22,23,24,25]. Most of these polymeric QACs are non-volatile, chemically stable [18], and show little toxicity toward human cells, all of which are major requirements for biomedical applications [3]. This makes these polymeric QACs prime candidates for use in medicines to fight infections, in food and drug manufacturing to avoid bacterial contamination, in water treatment to impede the growth of bacteria [26], and in water purification [27,28], and as catalytic materials [29]. These polymers also serve as potential drivers for overcoming antibiotic resistance. They are the most beneficial sanitizers and antiseptics [30,31] and are used for an assortment of medical purposes. Pathogenic-membrane active agents [32] predominately target the cytoplasmic (inner) membrane in bacteria and the plasma membrane in yeasts/fungi [17,33]. The following sequence of events can be accomplished when microorganisms are exposed to cationic polymers: adsorption, followed by diffusion/penetration of the polymer into the bacterial cell wall, reaction with the cytoplasmic membrane (lipid or protein), followed by membrane disruption, excel/leakage of intracellular low-molecular-weight material, degradation of proteins and nucleic acids, and wall lysis due to autolytic enzymes [3]. Disrupting the cytoplasmic membrane structures in bacteria and successive seepage of cytoplasmic elements results in the death of the bacteria cell [34,35]. Additionally, as surfaces of bacteria are negatively charged, the adsorption of polymeric cations was proven to be more efficient than the polymeric anions. However, it has been noted that polymeric QACs’ applications may cause resistance in microbes [5] and do not easily degrade in the environment as they are highly stable chemically [36,37]. Hence, there is a need for the advancement of biodegradable polymers with antimicrobial properties. In addition, the catalytic activity can be an effective method for the removal of azo dyes in water/wastewater effluents. Industries including textile and dye are the main sources of dyes released into the environment. Due to their eco-toxicity and persistence, these dyes were characterized as harmful materials. Therefore, polymeric QACs were observed to be very efficient in degrading the Congo red dye via photocatalytic activity using UV irradiation [38].

The overarching goal of this study is to prepare a highly viscous crosslinked cationic PRO-EPI composite using the polycondensation technique while exploiting the antimicrobial and adsorptive properties. Proline (PRO) was chosen as one of the components for PRO-EPI because PRO-based cationic polymers are stable and biodegradable, have the ability to complex with DNA molecules, and are non-toxic in nature [39]. The specific aims of this research were to (i) produce and characterize high viscosity PRO-EPI, (ii) assess the potential of PRO-EPI for inactivation of microorganisms, (iii) assess the PRO-EPI dosage for optimal inhibition of microorganisms, and (iv) explore the adsorptive properties.

## 2. Materials and Methods

All chemicals were used as received. L-proline (PRO), epichlorohydrin (EPI), ethylenediamine (ED), sodium hydroxide (NaOH), hydrochloric acid (HCl), sodium borohydride (NaBH_4_), sulfuric acid (H_2_SO_4_) and Congo red dye (CR) were purchased from Sigma-Aldrich (St. Louis, MO, USA). Nutrient Agar was acquired from Oxoid LTD (Hampshire, UK), and Fresh Mueller Hinton Broth was purchased from Sigma-Aldrich (St. Louis, MO, USA). Deionized water (≤18 MΩ) was used to prepare all aqueous solutions. The process for the synthesis of PRO-EPI was reported earlier [40].

### 2.1. Preparation of the Cationic Polymer

**Figure 1:**
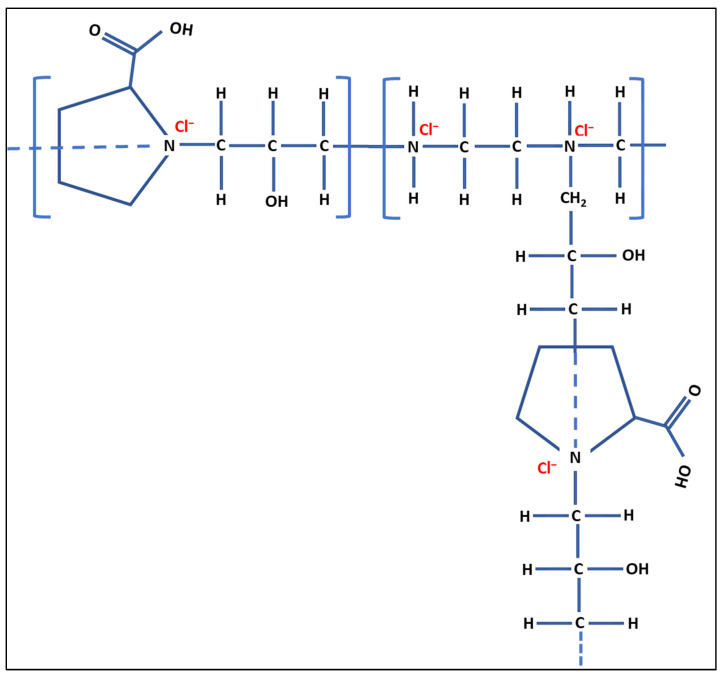
Schematic for formation of PRO-EPI.

In brief, polymerization was carried out in a three-necked round bottom flask containing a condenser and magnetic stirrer. Purified nitrogen was passed through the vessels for approximately 20 min. High viscosity crosslinked cationic PRO-EPI composite was prepared using polymerization of epichlorohydrin, followed by amination with proline and ethylenediamine. In brief, L-proline (3.55 g) along with distilled water and epichlorohydrin were mixed and stirred in the glass reactor at 25 °C, followed by the dropwise addition of ethylenediamine into the reactor. The temperature was then gradually increased to 70 °C, and the reaction was carried out for 1 h. The temperature of the mixture was further raised to 90 °C, and thereafter, an excess amount of epichlorohydrin was mixed, and the reaction was allowed to continue for a day in order to obtain highly viscous crosslinked composites. Concentrated sulfuric acid (H_2_SO_4_) was used to terminate the reaction. The details of the synthesis process are described elsewhere [41]. A plausible schematic of the formation of PRO-EPI is shown below (Figure 1). A detailed description is presented elsewhere [40].

### 2.2. Characterization Studies

The PRO-EPI was characterized by 1H-NMR (Bruker (400 MHz) spectrophotometer, Bruker, Billerica, MA, USA). The morphology of the PRO-EPI was analyzed by SEM (ZEISS EVO HD 15 Environmental Scanning Electron Microscope, Zeiss, Oberkochen, Germany). Functional groups of the PRO and PRO-EPI were studied using a Varian 800 FT-IR spectrophotometer (Varian Inc., Palo Alto, CA, USA). The viscosity of PRO-EPI was determined using Brookfield Viscometers (AMETEK Brookfield, Hadamar-Steinbach, Germany). A Zeta-Sizer particle size (ZEN 3600, Malvern Instruments, Malvern, Worcestershire, UK) instrument was used to measure the zeta potential of the PRO-EPI. UV–vis spectrophotometry characterization of the PRO-EPI was carried out using a UV–vis spectrophotometer (Lambda 7505 Model, Perkin Elmer, Shelton, CT, USA) with a scan range of 300–700 nm. Thermogravimetric analysis (TGA) of PRO-EPI was carried out using a TGA Q500 (TA Instruments, Elstree, UK). The DSC/TGA study was conducted at a heating rate of 10 °C/min in the range of room temperature to 800 °C. The viscosity of the sample affects the microbial resistance of the polymer. A Brookfield Viscometer (model no. CAP 2000, Spindle 05, AMETEK, Middleboro, MA, USA) was used to measure the viscosity measurement of the PRO-EPI at 750 rpm and 25 ± 0.05 °C. The experimental viscosity of the PRO-EPI was 940 cps. An aqueous solution of NaBH_4_ (0.5 mL, 15 mM, ice cold) was mixed with a stock solution of CR azo dye (1.8 mL, 10^−5^ mM) in a quartz cuvette and PRO-EPI (25 µL) was added. The final volume of the mix was adjusted to 3 mL with DI water.

### 2.3. Antimicrobial Activity

Fresh Nutrient Agar media was prepared as follows: 28 g was weighed out into 3 separate one-liter glass bottles filled to the one-liter mark with distilled water and mixed until the powder completely dissolved. The bottles were sterilized by autoclaving for 15 min at 121 °C. The resultant agar was transferred into plates for solidification.

#### 2.3.1. Preparation of Reagent: Microplate Alamar Blue Assay (MABA)

Resazurin powder (0.2 g) was dissolved in 10 mL of distilled water (autoclaved), and the dye solution was rigorously mixed and instantly covered using an aluminum foil to avoid exposure to light [41,42].

#### 2.3.2. The Preparation of the Nutrient Broth

Mueller Hinton Broth was prepared following the manufacturer’s instructions. Nutrient Broth powder (23 g) was weighed into a 1 L glass bottle and filled with distilled water. The solution was mixed until the broth was completely dissolved. Followed by sterilization in Bijou bottles by autoclaving for 15 min at 121 °C. The microbial cultures of *S.aureus*, *E. coli*, and *P. aeruginosa* were spread on nutrient agar slopes at 4 °C and subcultured onto blood agar plates for 24 h prior to usage.

#### 2.3.3. Minimum Inhibitory Concentrations (MICs)

Serial dilution techniques were used to determine the MICs of the PRO-EPI against both the Gram-positive (*S. aureus* (ATCC 29213) and Gram-negative (*E. coli* (ATCC 25922) and *P. aeruginosa* (ATCC 27853) bacteria. Test tubes were inoculated using a bacterial suspension (10^6^ CFU/mL) and incubated at 37 °C for 24 h. The MICs were the least concentration of the extract, which did not result in any visible growth of bacteria in comparison to extract-free broths. Each extract was tested in triplicates (unless specified) with each bacterium. The activities of the synthesized PRO-EPI was examined using a Microplate Alamar Blue Assay (MABA) with 96-wells microplates, including a positive control that consisted of antibiotic and a growth control which was pure culture broth. Then 20 μL of different concentrations of PRO-EPI was added to two neighboring wells except for the positive and growth control sample wells. The total volume in each well reached 200 µL with the addition of 20 μL of Alamar Blue. The final concentrations of the examined PRO-EPIs were 256, 128, 64, 32, 16, 8, 4, 2, and 1 µg/mL. After incubation, data were recorded as MICs (minimum inhibitory concentrations) of each PRO-EPI that completely subdued the growth of microorganisms. PRO-EPI was assessed for antimicrobial properties against *E. coli* (ATCC 25922), *P. aeruginosa* (ATCC 27853), and *S. aureus* (ATCC 29213). Ciprofloxacin and Nalidixic were used as positive controls; an examination of the data showed that the polymer indicated no inhibitory properties against *staphylococcus*. However, PRO-EPI showed that the MICs of 128 µg/mL were effective against Gram-negative bacteria (Table 1). The activities of PRO-EPI composites (indicated as M5 in Figure S1, see Supplementary Materials) were compared with standard antimicrobial drugs (Ciprofloxacin and Nalidixic acid) as antimicrobial standards.

## 3. Results and Discussion

**Figure 2:**
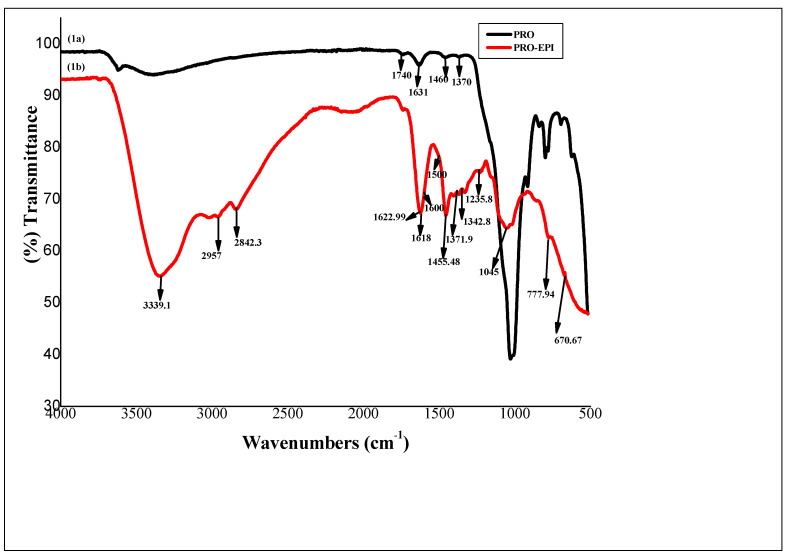
FT-IR spectra of (1a) PRO and (1b) PRO-EPI.

The FT-IR spectrum for pure PRO was characterized using the bands occurring at 1460 cm^−1^ and 1370 cm^−1^ (vibrations of the CH_2_ group), 1740 cm^−1^ (C=O band stretching vibration), and 1631 cm^−1^ (υC=O band) (Figure 1). A strong band appears at 1620 cm^−1^ assigned to the –COOH functional group of the PRO [43]. The following bands were observed for the PRO-EPI: C-H stretch bands at 2842.3 cm^−1^, C–H bands at 1455.48 cm^−1^, alcohol O–H stretch at 3339.1 cm^−1^, carboxylic acid O–H stretch at 2960 cm^−1^ and a C–O stretch at 1235.8 cm^−1^ (Figure 2). Weak vibrational bands were observed around 1500 cm^−1^ to 1600 cm^−1^, Which may be due to the C–H bend from the polymer proline [40]. The additional new band at 1045 cm^−1^ in PRO-EPI indicates the occurrence of aromatic C–N vibration of quaternary ammonium salts. Peak observed close to 1618 cm^−1^ maybe the result of bending and stretching of the quaternary ammonium group [44]. Moreover, the peaks below 3000 cm^−1^, specifically the very weak additional band noticed at 2957 cm^−1^, maybe related to asymmetrical elongating of CH_2_ as well as –NH_3_^+^ units linked to PRO-EPI, as reported in the literature [45]. FTIR data indicate that the PRO-EPI containing functional groups of quaternary ammonium and –COOH of the PRO were successfully prepared.

^1^H-NMR spectrum for the PRO-EPI [40] shows shifts at 1.95 ppm and 3.40 ppm due to methyl shifts. Chemical shifts at 4.10 and 2.40 ppm correspond to carboxylic protons and alcohol and CN-H proton, respectively [41]. The signals at 3.17 and 3.32 ppm are due to the methylene protons [46] connected to the quaternary nitrogen. The signal at 1.80 ppm was due to the methylene proton from ED [46], and the signal at 3.2 ppm was the quaternary ammonium protons as a sharp singlet [46].

The morphology of the PRO-EPI surface was studied using SEM [40]. SEM image of PRO-EPI shows the compact sheet with rough surface morphology, showing the amorphous nature of the particle. The polymer acts as a film, linking particles and reducing vertical grouping, and resulting in further optimization of adsorption due to increased exposure to the surface area of the composite particles [40]. PRO-EPI particles ranged from 20 to 216 μm, thereby providing an adequate surface area for the adsorption of microorganisms. Zeta potential of the PRO-EPI showed a positive charge of +6.94 mV [40]. The three peaks observed may be due to the different sizes of the dissolved polymer. This cationic charge could be due to the formation of a polymeric quaternary ammonium segment in PRO-EPI [40].

DSC/TGA data of the polymeric-quaternary ammonium salts (QAS) in the nitrogen atmosphere are shown in Figure S1. Results revealed that high viscosity/molecular weight polymers required high temperatures for degradation. Experimental data showed that the degradation temperature of PRO-EPI was over 201 °C, although some degradation was observed at lower temperatures. The slight weight loss of the sample below 201 °C could be due to the desorption of any residual water left in the polymer. PRO-EPI had a higher degradation temperature (201 °C) when compared to other cationic polymers (dimethylaminoethyl methacrylate-benzyl chloride/dodecyl bromide/hexadecyl bromide) (190 °C), which may be because of higher molecular weight [47]. Due to its heat stability, this polymer may be useful in many fields, including but not limited to biomedical devices, water treatment, functional fabrics, and others [48]. Figure S1 (see Supplementary Materials) shows that the PRO-EPI weight decreases from the beginning, implying PRO-EPI is hygroscopic, which can be due to the quaternary ammonium ion group [47]. The initial stage of degradation was due to the removal of water and the disintegration of the QAS groups. The second stage is due to the C–N–C polymer chain destruction. This could be due to the partially ionic nature of C–N–C moiety and the high bond separation energy of the C–N bonds. Therefore, the C–N–C skeleton in the quaternary PRO-EPI slows down the degradation and increases the thermal stability due to chain length. Hence, we can conclude that the PRO-EPI composite exhibits better thermal stability. The DSC thermogram appearing as an endothermic peak at 257.14 °C may correspond to the glass transition temperature (Tg) [41]. The higher Tg was due to its adamantine pendant groups and crosslinking that hinders rotation of chain segments. In addition, higher viscosity/molecular weight can lead to complex, long-range chain entanglements and restrict the scope for segmental rotation.

### 3.1. Evaluation of Antimicrobial Activity of PRO-EPI Composite

PRO-EPI is composed of QACs that contain a positive charge in the backbone along with methyl pendant functional groups (hydrophobic segment). Several factors influence the activity, the most important being the cationic nature of the composite. This composite first adsorbs onto the membrane, then inserts the hydrophobic moieties into the lipid bilayer of the microbe, resulting in membrane structure damage [49,50]. It possesses two functional characteristics: (a) multiple pendant chloromethyl groups that probably react with Gram-negative microorganisms and (b) cationic amino groups that reduce the charged barrier between polymer and the organism. The most important characteristic is the surface charge of the polymer, which influences the strong binding onto the cell membrane [51]. Since PRO-EPI is positively charged and *E. coli* and *P. aeruginosa* are anionic microorganisms, electrostatic interaction plays a vital role in the adsorption process [51]. The quaternary amine cationic groups (–N^4+^, –NH^4+^) can be used to attach to the microorganism by an electrostatic interaction via covalent bonding. These ammonium functional groups escalate the cationic nature of PRO-EPI, thereby facilitating stronger microbe surface-binding capability, which effectively inhibits further growth of microorganisms. The first step of the CR degradation process involved the breakdown of the –N=N– bonds. Additionally, the band at 338.08 nm, which indicates the “benzene-like” structures of CR, was deconstructed to a certain extent during catalytic reaction [38]. Hence, it may be concluded that CR decolorization and degradation took place on the PRO-EPI catalyst in the NaBH_4_ solution.

The cationic structure and the adsorption of microorganisms on the antimicrobial agent play a vital role in antimicrobial activity [51]. The activity of QACs was affected by multiple factors, including their structure, hydrophilic and lipophilic-hydrophobic balance, spacer distance between the active sites [52,53], the structure of bacteria cells, number of nitrogen atoms present, and the diffusivity of antimicrobial agents [48]. Table 1 shows the MICs of both types of microorganisms against PRO-EPI. It was evaluated by measuring the MICs of PRO-EPI. It was tested against *E. coli*, *P. aeruginosa,* and *S. aureus* microorganisms. Their antimicrobial properties were compared with the positive control and growth control (Figure S2, see Supplementary Materials).

MICs of both samples were 128 µg/mL for Gram-negative bacteria but not for Gram-positive bacteria, as observed by their activities. The higher activity of PRO-EPI at a MIC value of 128 µg/mL may be due to the presence of hydrophobic moieties such as methyl pendant reactive functional groups [19,20]. In addition, the high viscosity and cationic groups (–N^4+^, –NH^4+^) present in PRO-EPI promoted the activity, which is further improved by the presence of hydroxyl (OH^−^) groups. The positively charged hydrogen on an –OH groups have the potential to attract the Gram-negative cell wall of the microbial membrane [19]. It may therefore be concluded that the MICs are strongly influenced by the polymeric cationic structure. The microbial resistant characteristics of Gram-negative species are lower compared to Gram-positive bacteria. This may be a result of electrostatic interaction balance, which can be controlled by the charge neutralization process. For example, *E. coli* and *P. aeruginosa* are negative, whereas PRO-EPI is positively charged; hence, it is expected that a much stronger surface-binding capability [51] results when compared with *S. aureus,* disabling further cell growth. The MICs values of other commonly used pyridine-based and quaternary ammonium polymeric compounds of MICs were 200 and 59–156 µg/mL, respectively [54,55,56]. MICs of PRO-EPI in this study (128 µg/mL) were similar to those reported elsewhere but resulted in better antimicrobial activities. The negative bacteria showed the highest affinity and higher level of susceptibility to PRO-EPI.

The activity increased as a result of the number of quaternized nitrogen atoms in the main chain. Moreover, the electrostatic interaction could result in adsorption of PRO-EPI onto the cell surfaces by and membrane damage may be caused by subsequent leakage of low molecular weight compounds (K^+^ ions and other cytoplasmic constituents) [57,58]. In addition, the smaller particle size (observed in SEM) may enhance the surface area of the PRO-EPI, providing a favorable environment to adsorb microbes in aqueous solutions. When *S. aureus* was tested with the same MICs (128 µg/mL), there was no activity, indicating that PRO-EPI was effective only for the Gram-negative bacteria. This was expected, given the cell membrane is enclosed in a thick and porous cell wall that permits small molecules to diffuse. Since *S. aureus* contains thicker peptidoglycan, it was predicted that high viscosity/molecular weight polymers would not readily diffuse through this cell wall as in the case of the Gram-negative *E. coli* [59], which have a thinner peptidoglycan layer. Factors such as higher viscosity of the PRO-EPI inhibited penetration through the cell wall of *S. aureus* [59]. The higher viscosity/molecular weight, diffusivity, mobility, attraction, and ionic interaction of larger chains do not facilitate adsorption and contribute to the ineffective binding to the membrane’s surface [60]. Consequently, PRO-EPI could not diffuse freely into the cytoplasmic membrane and, hence, could not be permeable through the cell wall. Ikeda et al. [61] reported that antimicrobial properties declined abruptly when the molecular weight increased. However, in spite of its lower penetration and neutral interactions with the membrane, we assume that antimicrobial activity was driven by electrostatic interactions. Anionic lipid composition in microbial membranes differs among bacteria; however, it is estimated to be <20%. Interactions between positive charges in the polymers and negative charges dispersed in the microbial membrane play a crucial role in antimicrobial activity [59]. The PRO-EPI composite has a reduced ability to interact and contact due to its poor diffusivity. This might decrease the penetration of PRO-EPI into the cell wall. As a result, the PRO-EPI exhibited no bacterial activity against *S. aureus*. On the other hand, the structure of *S. aureus* allows the PRO-EPI to collect at the outer and inner membranes. These findings could potentially explain why EPI-PRO inactivates *E. coli* and *P. aeruginosa* but not *S. aureus.*

Spacer length between the active sites within the polymer is noticeably different, and structure configuration also influences microbial activity. The long chain increases the spacer length and hindrance between the polymer and the hydrophobic bacteria [62]. It is reasonable to conclude that the smaller spacer enhances the charge density/cationic; therefore, the hydrophilic chain segments may not outweigh the hydrophobic *S. aureus* bacterium [2], leading to diminished activity [46]. Additionally, the increased hydrophobicity of *S. aureus* strains may reduce attraction with the PRO-EPI antimicrobial agent, resulting in ineffective activity [62]. In summary, the data suggest that PRO-EPI is significantly active against Gram-positive bacteria compared to *S. aureus*. This could be partially due to the reduced hydrophobicity and enhanced negative surface charge on Gram-negative microorganisms. It was observed that Gram-positive bacterium cells are in contact with cationic polymeric QACs. D-alanylation of teichoic acids and the lysylation of phosphatidylglycerol reduce the negative charge of the cell surface and membrane, thus resulting in hindering the adsorption of the cationic antimicrobials [63].

### 3.2. Adsorption of CR Azo Dye with PRO-EPI

**Figure 3:**
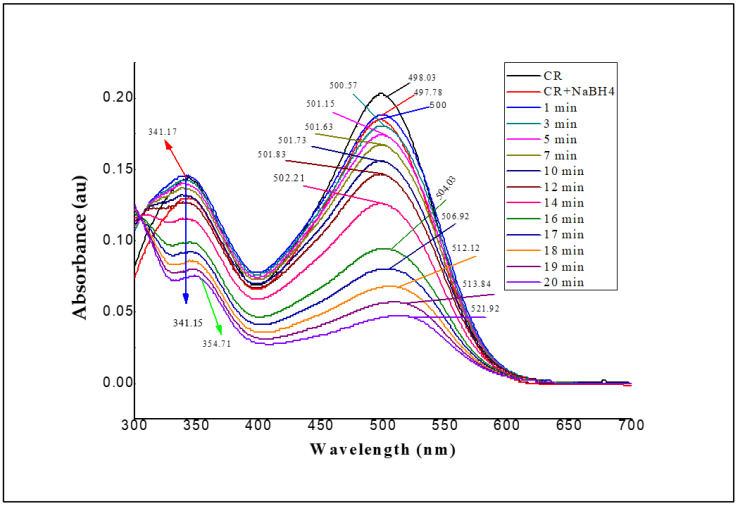
UV-vis spectra of CR, CR + NaBH_4_, and PRO-EPI loaded CR + NaBH_4_, CR control, and CR + NaBH_4_. Control was similar to CR and CR + NaBH_4_ (data not shown here).

The mixture of NaBH_4_ and Azo dye was agitated, and the reaction was monitored using a UV-vis spectrophotometer (every two minutes) in the 300–700 nm range at room temperature (Figure 3). Control experiments with CR over time without the addition of PRO-EPI did not show any removal (data not shown here).

Figure 3 shows the adsorption of PRO-EPI with CR in industrial wastewater obtained from textile industries. One chemical reaction was selected to evaluate the activity of PRO-EPI that qualifies as a model reaction, i.e., the reduction of CR by NaBH_4_ [64]. CR possesses two main characteristics: one main band with its maximum absorption at 498.03 nm (azo bonds) and another band located at 341.17 nm (benzene, naphthalene-based rings), as observed in Figure 3. A high-intensity absorption peak at 498 nm was observed for CR and CR+NaBH_4_ solutions in the absence of PRO-EPI. This indicates that NaBH_4_ was not independently able to degrade CR molecules directly. It was observed that λ_max_ was 498.03 nm and the absorbance intensity gradually decreased with time. A notable difference was observed in the absorbance of CR at 0.05 a.u at 20 min reaction time interval. This intensity decreased over a shorter period. Since the most prevalent peak wavelengths shifted during the adsorption, we can assume that there was a variation in the polymer size as indicated by the broad asymmetrical absorption peaks.

Initially, the adsorption process occurred slowly as the active sites on the catalyst surface were shielded by CR due to the higher dye concentration [38]. However, as time progressed, a notable reduction in absorbance of the CR was noticed. This may be due to the availability of active sites on the catalyst surface and the screening effect [65,66]. A huge reduction in absorbance was observed in the final stage (20 min), indicating catalytic adsorption. During adsorption, the absorbance values and peak intensity diminished while there were no longer any specific peaks at 498.03 nm. This was due to the priority of cleavage of the azo groups in the catalytic activity, resulting in the rapid disappearance of chromophores in the CR molecule [38]. The adsorption process was vigorous as time progressed, and the color of the CR dye faded gradually due to the elimination of azo compounds that are responsible for the colorization. In addition to the decolorizing effect, the breakdown product of the absorbance in the 341.17–354.71 nm range shows the aromatic fragment degradation in the dye molecule and its intermediates [38]. It is worth mentioning that decolorization and also the degradation of CR took place on PRO-EPI catalyst in NaBH_4_ solution. The chemical reaction that converts the CR to the sulfonated azo and bis phenyl groups seems to be well controlled.

The absorption spectra of CR degradation at different times were investigated in the presence of PRO-EPI to determine the adsorption rate constant with respect to time-dependent absorbance. The rate constant (R^2^) was determined from the linear plot of ln A vs. reduction time (Figure S3, see Supplementary Materials). The reaction rates were calculated by taking the logarithm values of absorbance (ln A) at 498 nm vs. time, and the plots showed a strong linear correlation. A total of 25 µL of PRO-EPI was added as a catalyst and demonstrated catalytic adsorption activity with a good rate constant value of 0.92 ks^−1^.

It should be noted that NaBH_4_ is toxic, though this effect could be reduced as the reaction mechanism produces sodium metaborate (NaBO_2_), which has lower toxicity [67]. As described earlier, polymeric QACs are being widely applied due to their low toxicity. PRO-EPI behaves similarly and may be used as a catalyst. Therefore, decolorization and degradation of CR were evident on PRO-EPI in the NaBH_4_ solution. The CR adsorption was very fast as monitored by UV-vis spectrometry.

## 4. Conclusions

Performance of PRO-EPI-based composites tested with two types of microorganisms under identical conditions was reported. PRO-EPI catalyst prepared using a simple polycondensation process was effectively applied for *E. coli* and *P. aeruginosa* degradation and simultaneous decolorization of CR. Electrostatic interactions played a major role in the adsorption process. MICs obtained in this study were much lower than the pyridine-based and quaternary ammonium polymeric compounds and also resulted in better antimicrobial activities. Negative bacteria showed high affinity and a higher level of susceptibility to PRO-EPI. The profile of the peaks co-evidenced in the UV-vis spectrometry confirmed the removal of CR. It was only necessary to employ 25 µL of PRO-EPI catalyst to achieve the adsorption of the azo compounds tested. Based on the results of this study, polyamine-based PRO-EPI could be an attractive catalyst to facilitate degradation in dye polluted industrial wastewater.

## Figures and Tables

**Table 1:** Antimicrobial activities of polymer (M5): minimum inhibitory concentrations (MICs).

Polymer (PRO-EPI) Code	*Staphylococcus aureus* (µg/mL)	*Pseudomonas aeruginosa* (µg/mL)	*Escherichia coli* (µg/mL)
M5	-	128 ± 3.5	128 ± 2.5

**Note:** Antimicrobial drugs: Ciprofloxacin and Nalidixic acid. (-) had no antimicrobial effect.

## Data Availability

Not applicable.

## Supplementary Materials

The following supporting information can be downloaded at: https://www.mdpi.com/article/10.3390/polym14142797/s1, Figure S1: DSC-TGA profiles of PRO-EPI; Figure S2: Sample microtiter plate showing the MICs for tested compounds against Gram-negative microorganism on *E. coli*, *P. aeruginosa* and *S. aureus*. Note: Top to bottom concentrations are 256, 128, 64, 32, 16,8, 4, and 2 ug/ml. Top left: *E. coli*; Top right: *Pseudomonas*; Bottom: *Staphylococcus*. Blue color represents inhibition and pink color represents growth.; Figure S3: UV–vis spectra absorption spectra kinetic plots of ln A (A = absorbance at 498 nm) versus time.

## Abbreviations

DLS	Dynamic Light Scattering
DSC	Differential Scanning Calorimetry
EPI	Epichlorohydrin
FTIR	Fourier Transform Infrared Spectroscopy
MIC	Minimum inhibitory concentration
NMR	Nuclear Magnetic Resonance
PRO	Proline
SEM	Scanning Electron Microscope
TGA	Thermogravimetric Analyzer
