# Supplementary material for: Synthesis and Characterization of High Viscosity Cationic Poly(Proline-Epichlorohydrin) Composite Polymer with Antibacterial Functionalities
Source: Polymers (Basel). 2022 Jul 8;14(14):2797. doi: 10.3390/polym14142797 (PMC9323103; doi:10.3390/polym14142797)
Supplement: Supplementary file 1 [file polymers-14-02797-s001.zip › polymers-1760115-supplementary.pdf]

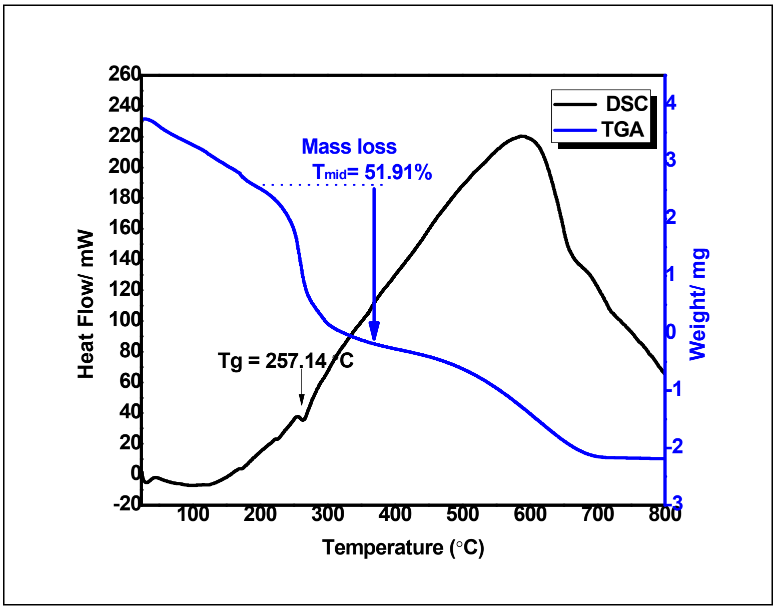

Figure S1: DSC-TGA profiles of PRO-EPI.

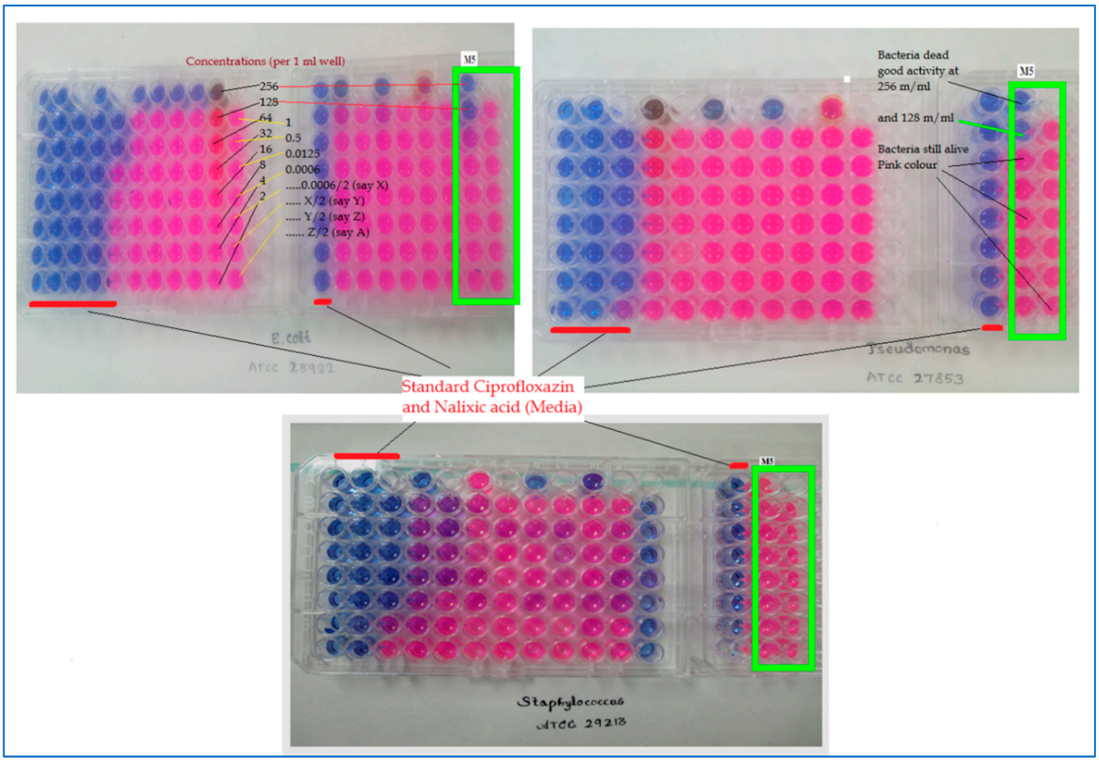

Figure S2: Sample microtiter plate showing the MICs for tested compounds against Gram-negative microorganism on E.coli, P. aeruginosa and S. aureus. Note: Top to bottom concentrations are 256, 128, 64, 32, 16,8, 4, and 2 ug/ml. Top left: E. Coli; Top right: Pseudomonas; Bottom: Staphylococcus. Blue color represents inhibition and pink color represents growth.

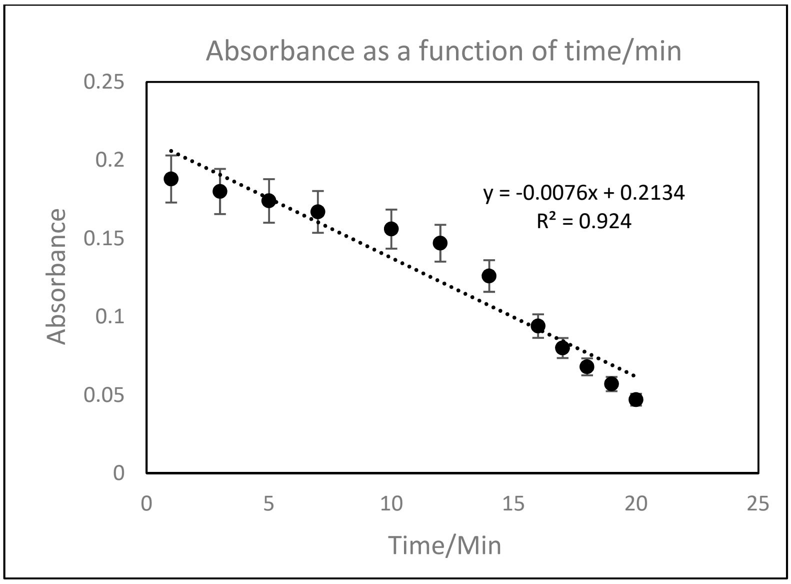

Figure S3: UV–vis spectra absorption spectra kinetic plots of ln A (A = absorbance at 498 nm) versus time.
